# Effect of a salt‐reduction strategy on blood pressure and acceptability among customers of a food concessionaire in Lima, Peru

**DOI:** 10.1111/1747-0080.12449

**Published:** 2018-07-16

**Authors:** Isabel A. Reynoso‐Marreros, Perlita K. Piñarreta‐Cornejo, Percy Mayta‐Tristán, Antonio Bernabé‐Ortiz

**Affiliations:** ^1^ Faculty of Health Sciences Universidad Peruana de Ciencias Aplicadas (UPC) Lima Peru; ^2^ Dirección de Investigación y Desarrollo Universidad Científica del Sur Lima Peru; ^3^ CRONICAS Center of Excellence in Chronic Diseases Universidad Peruana Cayetano Heredia Lima Peru; ^4^ Faculty of Epidemiology and Population Health London School of Hygiene and Tropical Medicine London UK

**Keywords:** blood pressure, public health, salt, satisfaction

## Abstract

**Aim:**

Limited information exists regarding the implementation of salt reduction strategies on collective food services, such as restaurants and food concessionaires. The present study aimed to assess the effect of a salt reduction strategy on blood pressure levels and food acceptability among customers of a food concessionaire.

**Methods:**

A quasi‐experimental study with two phases was conducted. In the pre‐intervention phase, the amount of salt used in food preparation was determined. In the intervention phase, a reduction of 20% in salt added to food preparations was implemented. Four hedonic tests and two blood pressure measurements were performed before and after the intervention implementation using standardised techniques. In addition, an evaluation of uneaten food was conducted daily on all customers’ plates. Mixed linear regression models were generated to assess the effect of the intervention on blood pressure and acceptability.

**Results:**

A total of 71 workers were evaluated, mean age of 37.5 years, 57.8% females, who consumed the food of the concessionaire, on average, 4.4 (SD: 0.7) days per week. Systolic and diastolic blood pressure were reduced by 3.1 (*P* < 0.001) and 2.9 (*P* < 0.001) mmHg at the end of the study, respectively. The results of the hedonic tests and the uneaten food before and after the intervention did not vary significantly.

**Conclusions:**

The reduction of 20% of salt added to food from a concessionaire had a positive impact on the reduction of customers’ blood pressure without reducing food acceptability. This strategy could be implemented in other contexts.

## Introduction

Worldwide, the increase of blood pressure levels is mainly affecting low‐ and middle‐income countries,^1^ leading to increased risk of cardiovascular disease, including stroke, myocardial infarction and chronic kidney disease.^2^ There is strong evidence suggesting that dietary salt is one of the major causes of increased blood pressure, and a reduction in salt intake can lower blood pressure, thereby reducing its consequences.^3^, ^4^

The World Health Organization (WHO) recommends that healthy adults should consume <5 g of salt per day (2 g of sodium).^5^ The average salt consumption in different communities from several countries has been estimated between 6 and 12 g/day.^6^, ^7^ As a consequence, many international organisations have highlighted the importance of generating scientific evidence about salt reduction as a public health measure to tackle hypertension.^8^ In addition to interventions, public health strategies should include surveillance and reduction of sodium in food.^5^, ^9^ However, there is scarce evidence regarding the acceptability of interventions reducing the amount of salt added to foods, especially in Latin American countries. Moreover, limited information exists regarding the impact of a salt reduction strategy on collective food services, such as restaurants and food concessionaires.

The collective food services (i.e. food concessionaires) are private companies engaged in the preparation of meals in large quantities aimed at commercialising them in universities, schools, hospitals, etc. In Peru, the food concessionaires must ensure the food safety standards before selling to customers,^10^ but there is no regulation regarding food components and supplements, such as the salt added to food. As the number of individuals consuming food outside home is increasing, this makes feasible to incorporate salt reduction strategies in food collective food services to have a positive impact on health.^11^

Therefore, the aim of the present study was to evaluate the potential impact of a salt reduction strategy on blood pressure levels and food acceptability among customers of a food concessionaire in Lima, Peru. In addition, in secondary analysis, the effect of this intervention was also evaluated according to hypertension status at baseline.

## Methods

A quasi‐experimental study, with two phases, including a pre–post assessment, was conducted and reported using the TREND statement for non‐randomised controlled trials.^12^ In the pre‐intervention phase, the salt added to food during preparation was estimated and four hedonic tests (satisfaction surveys) and two blood pressure measurements were performed. During the intervention phase, the salt‐reduction strategy was implemented and four hedonic tests and two blood pressure measurements were also conducted. In addition, an evaluation of uneaten food on customers’ plates after lunchtime was conducted daily during the two phases of the study.

The present study was conducted in a private company in Lima, Peru. The selected population was administrative workers (i.e. those that perform office activities). Only individuals aged ≥18 years old and who consumed food from the concessionaire at least three times a week were invited to participate. The present study was performed in two consecutive phases:

### *Phase 1: Pre‐intervention*

This phase included all procedures to ensure the implementation of the 20% salt reduction intervention and comprised:

### *Weighing salt added to food preparations*

**Figure 1:**
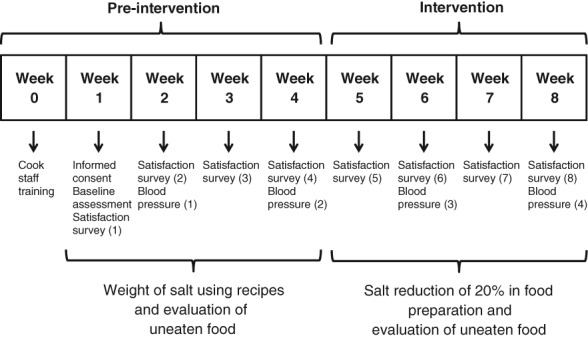
Flowchart of the phases of the study and assessments.

Initially (week 0, see Figure 1), the food concessionaire administrator and the main chef were informed about the aims and procedures of the study. The main chef and cook staff were trained to weigh salt added to food during preparation. The amount of salt used in preparations was estimated by weighing salt added to food during cooking. As the food concessionaire had a cycling program of menus repeated every four weeks, we decided that the weight of salt added to food during cooking would be conducted during four weeks in order to encompass the entire set of menus (weeks 1–4 of pre‐intervention phase, Figure 1). Weight estimations were recorded in a book to further modify the amount of salt added to food in recipes. Thus, salt added to main dish (i.e. meat, stews, etc.) and side dishes (i.e. rice, potatoes, etc.) was weighed using a balance scale (Miray, model BMR‐88, maximum weight: 10 kg, precision: 1 g).

### *Informed consent and baseline assessment*

During week 1 of the pre‐intervention study, all administrative workers fulfilling the selection criteria were invited to participate and informed consent was applied. Informed consent was focused on food acceptability and blood pressure measurements and not in the salt reduction strategy; therefore, participants were not aware that salt reduction would be implemented in the food concessionaire. After agreeing to be part of the study, individuals were given a short questionnaire containing the hedonic test as well as socio‐demographic information (age and gender), previous diagnosis of hypertension, anti‐hypertensive medication, smoking, alcohol consumption and physical activity as used in the Stepwise Approach to Surveillance of the WHO.^13^ In addition, a validated scale to assess satisfaction (hedonic test), as used in previous studies,^14^, ^15^ was applied in the study. Customer satisfaction was assessed by considering preferences and the mood of participants. A Likert‐type scale was applied with eight response options from ‘I extremely dislike’ to ‘I extremely like’.^16^ The hedonic test was applied to know about the acceptability of the meal of a day randomly selected. This evaluation was conducted weekly to all participants during lunchtime.

During the pre‐intervention phase, two blood pressure measurements were conducted. These assessments were carried out during the mornings of the second and fourth weeks of the study (Figure 1). A quiet office of a comfortable temperature was utilised to perform measurements. A period of five minutes was guaranteed before starting assessments. Systolic (SBP) and diastolic (DBP) blood pressure were evaluated using a previously validated automatic device (Riester, Ri‐Champion model, Jungingen, Ulm, Germany) in the left arm. Three different measurements were performed one minute apart, and blood pressure values were recorded.

### *Evaluation of uneaten food*

As part of the acceptability assessment,^17^, ^18^ an evaluation of uneaten food was also conducted. This was an indirect method to quantify food left by customers in dishes after lunchtime and used as a replacement of the direct weighing of uneaten food. Using standardised techniques, a scheme of the main and side dishes sold in the day was created, and the approximate area occupied by main dish (meat or stew) and side dish (rice, potato, etc) was calculated. A percentage of the uneaten food from each part of the meal was estimated to determine the amount of food left by the customer (potential options were: 0, 25, 50, 75 and 100%). This assessment was applied only to those who consumed the food in the dining room of the company and was conducted by direct observation when the clients left the dish tray at the end of the lunchtime. Assessment was conducted daily by two trained staff using a standardised template to guarantee an appropriate evaluation.

### *Phase 2: Intervention*

After determining the amount of salt added to food during preparation and completing the first four weeks of the study (Figure 1), the salt reduction strategy was implemented:

### *Standardisation of salt added to food*

A 20% reduction of salt added to food was implemented. This proportion of reduction was based on a previous study performed in a Peruvian bakery demonstrating that a reduction of 20% in salt content in bread was not detectable by customers.^19^ In addition, other international studies have also reported that this percentage of salt reduction might not affect food palatability.^20^, ^21^

A detailed list of recipes containing the amount of salt to be added in the intervention phase was created based on the information of the pre‐intervention phase. The weighted amount of salt to be added during food preparation was performed to calculate reductions and salt was placed in small bags to guarantee an appropriate process of cooking without adding extra amounts. The main chef, supervised by study staff and the concessionaire administrator, was responsible for adding salt to food. As part of the intervention, only the salt added to preparations was modified. The intrinsic salt of ingredients was not considered as this was not quantified. During the intervention, salt shakers were removed from the dining room to control the intake of additional salt.

### *Blood pressure, hedonic test and uneaten food evaluations*

Similar to the pre‐intervention phase, blood pressure and uneaten food were evaluated. Thus, four hedonic tests (weeks 5–8), two blood pressure assessments (weeks 6 and 8), and daily evaluation of uneaten food were conducted as in the pre‐intervention phase (Figure 1).

The exposure of the study was the intervention, the reduction of 20% in salt added to food preparations, in the concessionaire. The outcome variable was SBP and DBP assessed as numerical variables as well as scores of satisfaction surveys (hedonic tests) and results of the evaluation of uneaten food. Thus, blood pressure values were obtained from three consecutive measurements as explained before, and the average of the second and third assessments were used for analyses. Similarly, in the hedonic tests, a value was assigned to each of the response options from 1 for ‘I extremely dislike’ to 8 for ‘I extremely like’. As before, percentages were assigned to the proportion of food left by customer in the dish trays for each of the components of the meal (meat, stew and side dishes).

Other variables were also included in the analyses as potential confounders, including: sex (male *vs* females), age, education level (<12 *vs* ≥12 years), current smoking (yes *vs* no), alcohol consumption, based on the self‐reported frequency of alcohol consumed in the last 12 months and then split in two categories if consumed or not,^13^ physical activity (self‐reported as at least 30 minutes of vigorous physical activity per day, based on the question of a risk score^22^), and diagnosis of hypertension, defined as self‐report of a physician diagnosis of hypertension or SBP ≥ 140 or DBP ≥ 90 in the first study assessment (yes *vs* no).

The Power Analysis and Sample Size software (PASS 2008, NCCS, Utah, USA) was used for sample size calculation. Assuming a power of 80% and a significance level of 5%, 70 participants were required to detect a reduction of blood pressure, mainly in the SBP, of 3 mmHg with a standard deviation (SD) of 8 mmHg, and having at least four assessments in total with a correlation between measurements of 0.7.

Analyses were conducted using STATA 13 for Windows (StataCorp, College Station, TX, USA). Initially, a description of the study population at baseline was performed using means and SD for numerical variables and proportions for categorical variables. Mixed linear regression models with random intercept were created with two different levels: blood pressure assessments (or satisfaction survey scores) in the first level, and the study individuals in the second level. In the case of blood pressure, the crude model includes SBP or DBP (in mmHg) in each of the assessments as the outcome and the time (in weeks) as a categorical variable (2, 4, 6 and 8). Similarly, in the case of the hedonic test results, time was introduced into the model in the same way, but changes were weekly instead of every two weeks. The adjusted models also included age, sex, education level, current smoker status, alcohol consumption, physical activity and previous diagnosis of hypertension. Results were presented as coefficients and 95% confidence intervals (95% CI).

Finally, repeated‐measures analysis of variance (ANOVA) was used to assess whether changes in the uneaten food over time were significant or not. Calculations were conducted for each of the meal components (meat, stew and side dishes).

The protocol and informed consent forms were reviewed and approved by the Ethical Committee of the Universidad Peruana de Ciencias Aplicadas, Lima, Peru. Participants provided written informed consent, and all procedures were approved by staff of the food concessionaire.

## Results

A total of 71 workers, mean age of 37.5 (range: 24–60) years and 57.8% females, were enrolled in the study. The characteristics of the study population are detailed in Table 1. Individuals consumed the concessionaire food 4.4 (SD: 0.7) days a week on average, and 11 (15.5%) had hypertension, but only 7 (63.6% of those with hypertension) were aware of diagnosis and none of them reported being on treatment.

**Table 1:** Characteristics of the study population at baseline

Variables	Frequency
Sex	
Females, *n* (%)	41 (57.8)
Age, mean (SD)	37.5 (12.0)
Education level (year), *n* (%)	
<12	4 (5.6)
≥12	67 (94.4)
Food concessionaire use (days/week), mean (SD)	4.4 (0.7)
Current smoker, *n* (%)	7 (9.9)
Alcohol consumption, *n* (%)	65 (91.6)
Physical activity (at least 30 min/day), *n* (%)	43 (60.6)
Hypertension, *n* (%)	11 (15.5)

SD, standard deviation.

Changes in systolic and diastolic blood pressure before and after the salt reduction strategy are shown in Table 2. According to our multivariable model, the systolic blood pressure decreased by 2.5 mmHg (*P* < 0.001) in the sixth week of the study and 3.1 mmHg (*P* < 0.001) in the eighth week (two and four weeks after starting the intervention, respectively). Similarly, the diastolic blood pressure fell 1.9 mmHg (*P* < 0.001) and 2.9 mmHg (*P* < 0.001) in the same respective periods.

**Table 2:** Systolic and diastolic blood pressure changes over time: crude and adjusted linear mixed models

Time	Blood pressure levels					
	Systolic			Diastolic		
	Mean (SD)	Crude (95% CI)	Adjusted(a) (95% CI)	Mean (SD)	Crude (95% CI)	Adjusted(a) (95% CI)
Intercept	—	117.0 (114.3; 119.7)	116.1 (101.5; 130.7)	—	72.9 (71.1; 74.7)	76.2 (65.9; 86.6)
Week 2 (first blood pressure measurement)	117.0 (12.0)	Reference	Reference	72.9 (8.1)	Reference	Reference
Week 4 (second blood pressure measurement)	118.7 (11.3)	1.7 (0.5; 2.8)	1.7 (0.5; 2.8)	73.8 (7.8)	1.0 (0.1; 1.8)	1.0 (0.1; 1.8)
Week 6 (third blood pressure measurement)	114.5 (11.3)	−2.5 (−3.7; −1.4)	−2.5 (−3.7; −1.4)	70.9 (7.2)	−1.9 (−2.8; −1.1)	−1.9 (−2.8; −1.1)
Week 8 (fourth blood pressure measurement)	113.9 (11.8)	−3.1 (−4.3; −2.0)	−3.1 (−4.3; −2.0)	70.0 (7.6)	−2.9 (−3.8; −2.0)	−2.9 (−3.8; −2.0)

(a) Adjusted for age, sex, education level, current smoker status, alcohol consumption, physical activity and hypertension status.

CI, confidence interval; SD, standard deviation.

At the end of the intervention, the reduction of the systolic blood pressure was greater in hypertensive individuals (4.2 mmHg, *P*‐value = 0.006) compared to non‐hypertensive individuals (2.9 mmHg, *P* < 0.001). On the other hand, diastolic blood pressure decreased markedly among non‐hypertensive individuals (3.2 mmHg, *P* < 0.001) compared to those with hypertension (1.4 mmHg, *P* = 0.03).

The salt added to food during preparations decreased, on average, from 10.3 g (SD: 4.0) to 8.2 g (SD: 3.2) (i.e. about 2.1 g). Despite the 20% salt reduction in the food preparations, the score of the hedonic test did not change (between 5.8 and 6.1) during the eight weeks of the study. The multivariable model did not find significant changes in the score of the hedonic test before and after the intervention (Table 3).

**Table 3:** Impact of the salt reduction intervention on hedonic test results: crude and adjusted linear mixed models

Time	Hedonic test		
	Mean (SD)	Crude	Adjusted(a)
Intercept	—	5.9 (5.6; 6.1)	6.6 (5.5; 7.7)
Week 1	5.9 (1.5)	Reference	Reference
Week 2	5.8 (1.1)	−0.1 (−0.3; 0.2)	−0.1 (−0.3; 0.2)
Week 3	5.9 (1.0)	0.0 (−0.3; 0.3)	0.0 (−0.3; 0.3)
Week 4	5.8 (1.0)	0.0 (−0.3; 0.2)	0.0 (−0.3; 0.2)
Week 5	5.8 (0.9)	0.0 (−0.3; 0.2)	0.0 (−0.3; 0.2)
Week 6	5.9 (0.8)	0.0 (−0.2; 0.3)	0.0 (−0.2; 0.3)
Week 7	6.1 (0.9)	0.3 (−0.1; 0.5)	0.3 (−0.1; 0.5)
Week 8	6.1 (0.9)	0.3 (−0.1; 0.5)	0.3 (−0.1; 0.5)

(a) Adjusted for age, sex, education level, current smoker status, alcohol consumption, physical activity and hypertension status.

SD, standard deviation.

In addition, there was no variation over time in the uneaten food left by customers when using the repeated‐measures ANOVA test. Thus, for meat, stew and side dishes, the *P*‐value of the test was not significant (0.53; 0.21 and 0.71, respectively).

## Discussion

Our results show that it is feasible to implement a salt reduction intervention to decrease systolic and diastolic blood pressure in a sample of workers consuming meal from a food concessionaire company, without having an impact on food acceptability as demonstrated by a lack of change in the hedonic test and in the evaluation of uneaten food. In addition, our results suggest that this intervention can have an effect on those with hypertension, especially in systolic blood pressure.

Previous studies have reported that salt reduction is associated with lower levels of systolic and diastolic blood pressure.^23^, ^24^ However, there is scarce information assessing the potential impact of salt reduction strategies in blood pressure and acceptability in companies related to food sales such as food concessionaires, especially in low‐ and middle‐income countries. Consistent with our findings, a systematic review reported that a modest reduction in salt intake for ≥4 weeks caused relevant falls in blood pressure in both hypertensive and normotensive subjects.^23^ In addition, Sacks *et al*. assessed the potential effect of different levels of dietary salt in individuals with and without hypertension.^24^ They found that reducing salt intake decreased systolic blood pressure in 7.1 mmHg in normotensive individuals and 11.5 mmHg among subjects with hypertension. In this latter study, the intervention was combined with the dietary approach to stop hypertension diet with increased amounts of fruits and vegetables. Our intervention also shows a reduction in blood pressure as well, but only involved decreasing the salt added to food without changing the other ingredients used during preparation.

Different strategies have been used to change dietary patterns related to salt intake. For example, dietary education focused on salt reduction to reduce blood pressure at the population level has been studied. A report from Japan revealed that dietary education to decrease salt intake and increase micronutrients in healthy subjects could reduce systolic, but not diastolic, blood pressure levels.^25^ There is also evidence that dietary education might have a greater impact on population with increased salt consumption.^26^, ^27^ However, this kind of intervention could be expensive as human resources are needed to educate individuals, as demonstrated in a previous study in Finland.^28^ Although our intervention was focused on individuals working in a small company, only cook staff needed appropriate training to implement this intervention.

Other studies have reported that it is possible to reduce salt content in food without affecting acceptability. For example, salt content was reduced by 30% in vegetable soup for children and the elderly without affecting perceived saltiness.^29^ Similarly, a previous study found that a 20% reduction of salt content in bread in a bakery of Lima, Peru, was feasible without changing taste or affecting sales.^19^ Another study reported the feasibility of steadily reducing salt content in bread up to 25% without changing customer's acceptability.^21^ Alternatively, another strategy to reduce sodium intake is replacing it with substitutes as potassium chloride.^30^ These strategies are important as implementing interventions to decrease salt intake should also take into account the perceived saltiness and taste. This is especially true for food companies as sales can be affected by these interventions.

This is an inexpensive intervention comprising appropriate training and education to cook staff that can have an impact on blood pressure by reducing the salt added to food during preparation. As the most important fear of companies is affecting taste, and hence sales, salt reduction needs to be gradually performed. Our results are reassuring: it is feasible to reduce salt content in food in restaurants and food concessionaires without affecting acceptability.

It is necessary to reproduce our intervention in other contexts in different settings. This salt reduction strategy can be easily implemented in nurseries and shelters for children and nursing homes for the elderly, as well as similar institutions. These populations are especially vulnerable as there is evidence suggesting that salt consumption is higher than the recommended standards.^31^, ^32^ In addition, reduction of salt content in food for children may have beneficial effects on blood pressure in adolescence and adulthood.^33^ Similarly, among the elderly, a reduction of sodium intake can have a positive effect on blood pressure but also in cardiovascular pathologies,^34^ as well as also being considered as an alternative or an addition to hypertension medication.^35^ In addition, longer studies are needed to assess the long‐term effect of these kinds of interventions as well as to evaluate the implementation of greater reductions of salt content in food.

Our results suggest that it is possible to improve dietary patterns (salt reduction) in a population group. Results may be useful in supporting the implementation of similar strategies in similar and different contexts. However, the present study has some limitations. First, due to its quasi‐experimental nature, there was no control group; therefore, our results may be secondary to other unmeasured variables. However, our findings are similar to randomised clinical trials.^23^, ^24^, ^25^ In addition, confounders not assessed in the study may have influenced results. Nevertheless, our design with two measures before and after the intervention may address and control for confounding and regression to the mean, two of the most common problems in these type of studies.^36^ Second, selection bias may arise as the food concessionaire was selected by convenience. Together with the small sample size, this may affect the generalisability of results. Third, other non‐communicable pathologies, such as diabetes mellitus and hypercholesterolaemia, were not assessed, as self‐report is not a reliable method as many people are not aware of their condition.^37^, ^38^ In addition, potential confounders, such as obesity, as defined by body mass index, a well‐known risk factor for hypertension, and total diet were not included in the assessment. As participants were not aware of the salt reduction strategy, we do not believe this can bias results. Moreover, health assessment through questionnaires was conducted only at baseline and not in different times during the study. However, it is not expected to have significant changes in health over an eight week period. Finally, the size of the food portion served to customers was not assessed; however, the food concessionaire used standard serves periodically audited as part of the service they deliver to the client company.

In conclusion, the reduction of 20% of salt during food preparation carried out by a food concessionaire had a positive impact of systolic and diastolic blood pressure in customers without affecting acceptability. Similar strategies should be applied in similar and other contexts as it may have beneficial effects on individuals.

## Funding source

Antonio Bernabe‐Ortiz is a Research Training Fellow in Public Health and Tropical Medicine funded by Wellcome Trust (103994/Z/14/Z).

## Conflict of interest

The authors have no conflicts of interest to declare.

## Authorship

IAR‐M and PKP‐C conceived the idea of the study with the help of PM‐T and AB‐O. IAR‐M and PKP‐C drafted the first version of the manuscript with support of PM‐T. AB‐O performed the statistical analysis. IAR‐M and PKP‐C coordinated fieldwork activities. All authors participated in manuscript writing, provided important intellectual content and gave their approval of the version submitted for publication.
